# Takotsubo Cardiomyopathy in a Case of Intracerebral Hemorrhage: A Case Report

**DOI:** 10.7759/cureus.5711

**Published:** 2019-09-20

**Authors:** Raghav R Nagpal, Jeyhan B Dhabhar, Jaishree Ghanekar

**Affiliations:** 1 Internal Medicine, MGM Hospital & Medical College, Mumbai, IND

**Keywords:** acute cerebrovascular event, catecholamine excess, takotsubo cardiomyopathy, acute coronary syndrome, st elevation

## Abstract

Takotsubo cardiomyopathy may present like acute coronary syndrome and is characterized by reversible left ventricular (LV) apical ballooning in the absence of any significant underlying coronary artery disease. A 65-year-old lady presented to the ED with history of sudden onset left-sided weakness of body. Head CT scan was suggestive of right gangliocapsular intracerebral bleed with intraventricular extension. 2D Echo showed characteristic LV apical ballooning with hypokinesia and LV ejection fraction of 25%-30%. Diagnosing Takotsubo cardiomyopathy includes resolution in electrocardiogram (ECG) changes and reversible LV dysfunction on two-dimensional echocardiogram, and a normal coronary angiography.

## Introduction

Takotsubo cardiomyopathy often presents as acute coronary syndrome but the hallmark feature is reversible left ventricular (LV) apical ballooning in the absence of significant coronary artery disease. In Japanese, “tako-tsubo” means “fishing pot for trapping octopus,” and the left ventricle of a patient diagnosed with this condition resembles that shape [1]. Takotsubo cardiomyopathy is transient and typically precipitated by any acute stress event. 

## Case presentation

**Figure 1:**
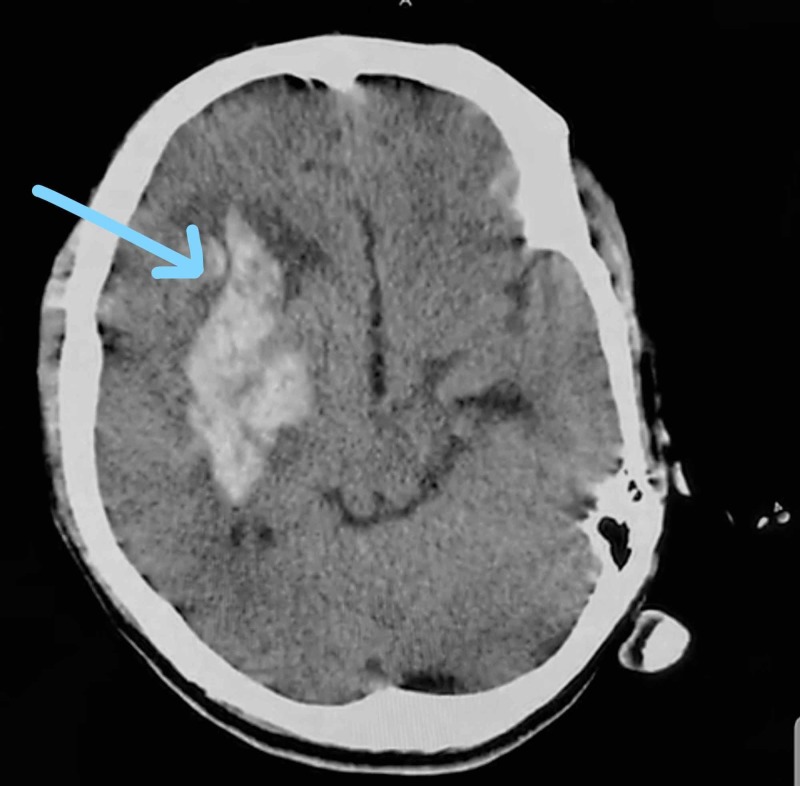
Head CT plain showing right large gangliocapsular hemorrhage.

 A 65-year-old lady with a history of sudden onset left side hemiparesis followed by drowsy disoriented state since two hours presented to the ED. On general examination she was tachycardic with a heart rate of 104 beats/min and a blood pressure of 160/90 mmHg. Neurological assessment was suggestive of power 0/5 in left upper and lower limb with left upper motor neuron facial palsy. No other neurological findings were present. Other systems were unremarkable. She was a known case of hypertension since two years on regular medications. No other known comorbidities were present. Routine blood investigations were all within normal laboratory limits. Head CT scan (Figure 1) was suggestive of right side gangliocapsular intracerebral bleed with intraventricular extension.

**Figure 2:**
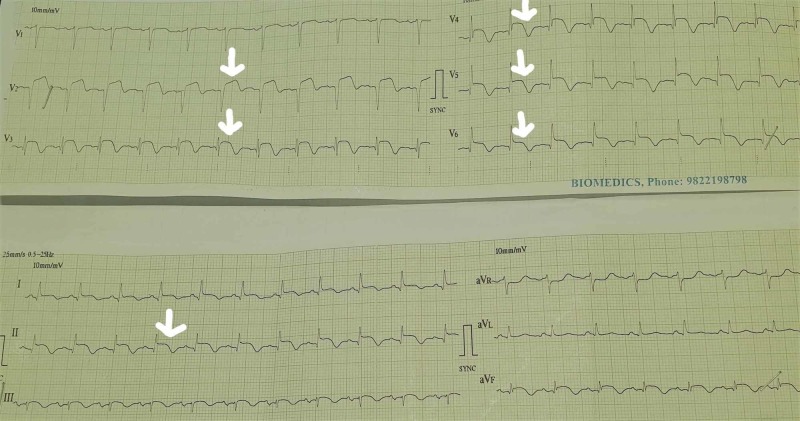
Electrocardiogram showing ST segment elevation v2-v6 , II, III, aVF leads.

Electrocardiogram (ECG) (Figure 2) showed sinus tachycardia, >2mm ST segment elevations in V2-V6, II, III, aVF., q waves v2-v6, no reciprocal changes. Cardiac enzymes (Table 1) were serially raised, Troponin-T at 0, 6, 12 h- 256, 898, 2144 IU/L respectively, and thereafter started declining. Subsequent ECGs over next few days showed complete resolution of ST segment elevations, disappearance of q waves, and appearance of deep T wave inversion in all precordial leads.

**Table 1:** Sequential cardiac enzyme levels.

	0 hours	6 hours	12 hours
CPK- MB	34	56	82
Trop-T	256	898	2144

**Figure 3:**
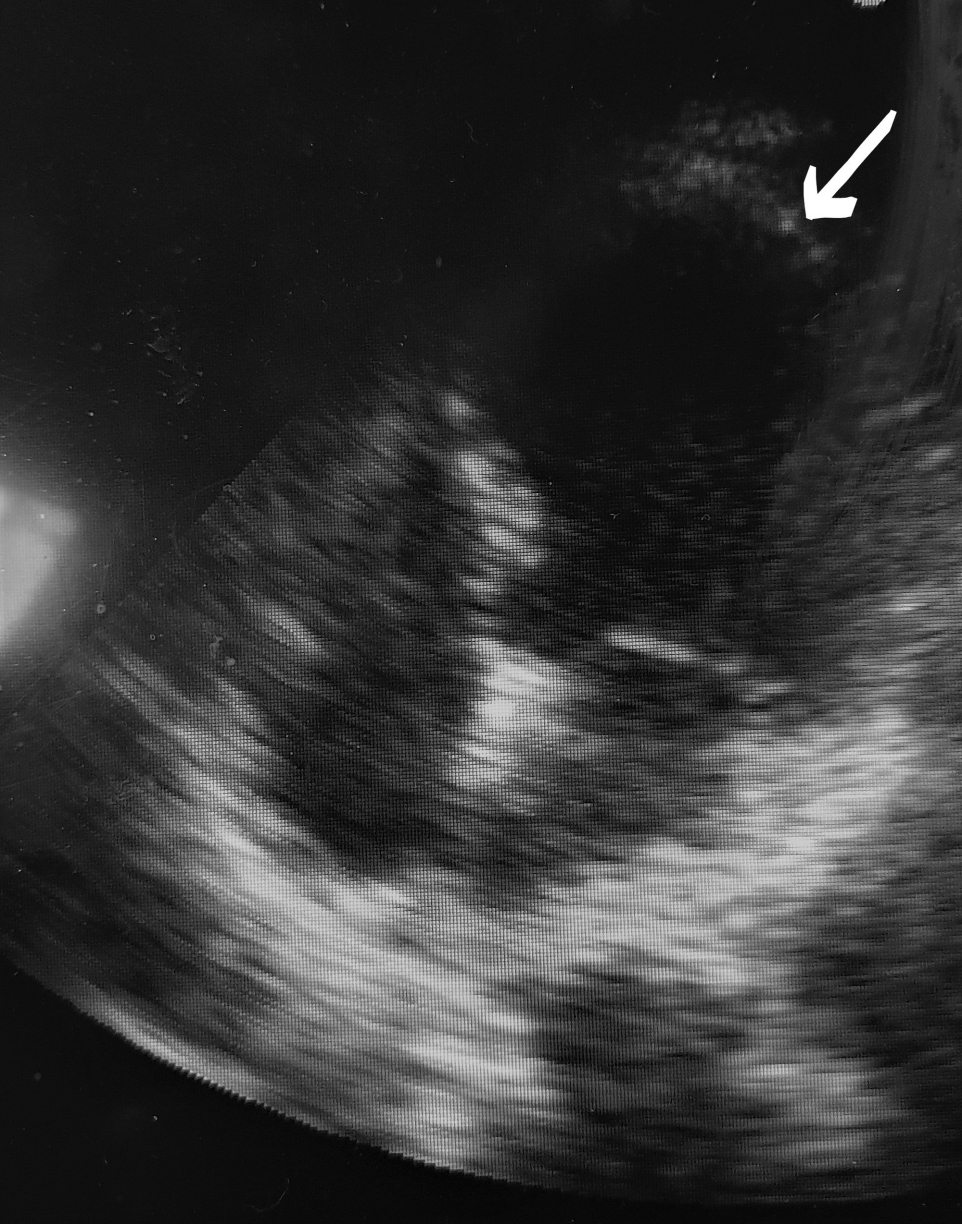
2D Echo showing characteristic left ventricular apical ballooning. 2D Echo, two-dimensional echocardiogram.

The two-dimensional echocardiogram (2D Echo) (Figure 3) showed characteristic LV mid segment hypokinesia with apical ballooning and LV ejection fraction of 25%-30%. No other abnormalities like valvular lesions, clot, vegetations or features of pulmonary artery hypertension were present. The ST-T changes on ECG, wall motion abnormality on 2D Echo were not limited to any particular coronary artery territory. There was resolution of ECG findings and 2D Echo findings on a review scan two weeks later with no residual regional wall motion abnormality on 2D Echo. All these findings support the diagnosis of Takotsubo cardiomyopathy, which has been previously described in literature to occur in association with acute cerebrovascular events. 

Patient was treated with beta blocker-tablet metoprolol 25 mg extended release preparation once daily for two weeks for Takotsubo cardiomyopathy, and for intracerebral hemorrhage with intracranial pressure reduction therapies, blood pressure control, mechanical ventilation, and other supportive care. The patient eventually expired on day 16 due to neurorespiratory complications.

## Discussion

Takotsubo cardiomyopathy was first recognized in Japan in the year 1990 and has since been defined as a separate entity. It shows preponderance in older adults and is more common in females than in males [2]. Of the 1750 diagnosed patients of Takotsubo cardiomyopathy in the International Takotsubo Registry, 89.9% were women and mean age was 66.4 years [3]. Similarly, in another review of 10 small prospective series, women accounted for 80%-100% of cases, with a mean age of 61-76 years [4]. A systematic review including 19 studies with a total of 1109 patients found that 39% of patients had emotional stressors and 35% had physical stressors [5].

In Takotsubo cardiomyopathy, the hypothesized mechanism is neurogenic stunned myocardium. This condition has been observed to occur during acute cerebrovascular accidents and during the catecholamine-induced cardiomyopathy in patients with pheochromocytoma [6], enhanced sympathetic activity appears to play a very important role in the pathophysiology of Takotsubo cardiomyopathy. Catecholamines have been shown to induce myocardial damage, and excessive stimulation of cardiac adrenergic receptors has led to transient LV hypo contractility in animal models [7]. ECG manifestations include ST-segment elevation or T-wave inversion, similar to acute coronary syndrome (ACS). The following changes occur commonly on ECG: (1) ST segment elevations and T inversions; (2) T inversions appear after ST elevation have subsided; (3) once T inversion appear, QT prolongation occurs; (4) there are no reciprocal change in limb leads; (5) Q-waves are reversible; (6) there is no atrioventricular block; and (7) any significant arrhythmia event is rare [8]. Other criteria for diagnosing Takotsubo cardiomyopathy includes resolution in ECG changes and reversible LV dysfunction on 2D Echo, and a normal coronary angiography [9].

We use the following proposed Mayo Clinic diagnostic criteria, all four of which are required for the diagnosis [10]:

●Transient reversible LV systolic dysfunction (hypokinesis, akinesis, or dyskinesis). The wall motion abnormalities are usually regional and extend beyond a single coronary artery distribution area; rare exceptions are the focal (within one coronary distribution) and the global type.

●Absence of significant coronary artery disease and angiographic evidence of acute plaque rupture. The diagnosis of stress cardiomyopathy can still be made in the presence of coronary artery disease if the wall motion abnormalities are not in the distribution of the diseased coronary vessel. This criterion is made as some patients with stress cardiomyopathy can have concurrent coronary artery disease (15.3% in the International Takotsubo registry).

●New onset electrocardiographic changes - ST elevations/T wave inversions or elevations in cardiac troponin values.

●Absence of pheochromocytoma or myocarditis.

In our patient, coronary angiography was not done due to poor neurological status and overall prognosis of the patient. 

## Conclusions

In patients presenting with stroke/acute brain injury with ST-T changes on ECG, a high clinical suspicion should be kept for Takotsubo cardiomyopathy as it can mimic acute coronary syndrome. The occurrence of Takotsubo cardiomyopathy is being increasingly recognized across the world and our understanding of this unique entity continues to evolve. 
